# A Meta-Analysis: Coronary Artery Calcium Score and COVID-19 Prognosis

**DOI:** 10.3390/medsci10010005

**Published:** 2022-01-21

**Authors:** Kevin Kyungmin Lee, Osman Rahimi, Charlie Kyungchan Lee, Amaan Shafi, Dalia Hawwass

**Affiliations:** 1 Department of Internal Medicine, University of Nevada Las Vegas, Las Vegas, NV 89102, USA; osman.rahimi@unlv.edu; 2 Department of Mathematical Sciences, Eastern University, St. Davids, PA 19087, USA; charlie.lee@eastern.edu; 3 Department of Cardiology, University of Nevada Las Vegas, Las Vegas, NV 89102, USA; amaan.shafi@unlv.edu (A.S.); dhawwass@gmail.com (D.H.)

**Keywords:** atherosclerosis, coronary artery disease, COVID-19

## Abstract

**Background**: Multiple studies have investigated the correlations of mortality, mechanical ventilation, and intensive care unit (ICU) admissions with CAC scores. This analysis overviews the prognostic capability of CAC scoring in mortality, mechanical ventilation, and ICU admission for hospitalized COVID-19 patients. **Methods**: Online search was conducted on PubMed, Cochrane Library, and Scopus from inception to 22 November 2021 to identify studies involving CAC scores in relation to ICU admission, mechanical ventilation, and death rates. **Results**: A total of eight studies were analyzed. In the absence of CAC group compared with the presence of CAC score, there was an increase in mortality in the presence of CAC (RR 2.24, 95% CI, 1.41–3.56; *p* < 0.001). In the low CAC group and high CAC group, high CAC group had increase in mortality (RR 2.74; 95% CI, 1.94–3.86; *p* < 0.00001). There was no statistical difference in outcomes of mechanical ventilation and ICU admission between any of the groups. **Conclusion**: This meta-analysis strictly examined the outcomes of interest in death, mechanical ventilation, and ICU admission while comparing the CAC scores in patients with COVID-19. Given these findings, CAC scoring can aid in stratifying patients, thus allowing earlier interventions in rapidly developing illnesses.

## 1. Introduction

The association between coronary artery calcification (CAC) and atherosclerotic disease is well known. The CAC score was created to help guide physicians to better calculate atherosclerotic risk in non-invasive nature to stratify risk for major adverse cardiac events. Currently, Agatston scores >100 generally delineate at least a moderate disease [1]; however, ordinal calcium scoring may also be applied to examine the extent of coronary calcifications. This analysis overviews eight studies on the prognostic capability of CAC scoring in coronavirus disease 2019 (COVID-19) hospitalizations.

Multiple studies have investigated the effects of CAC scores in hospitalized patients with COVID-19 as a potential prognostic marker. There has been some evidence of correlations in increased risk of mortality, mechanical ventilation, and intensive care unit (ICU) admissions. These studies echo that early CAC assessment can aide physicians to better triage high-risk patients. The direct and indirect effects of COVID-19 including arrhythmia, acute myocardial injury, Takotsubo syndrome, myocarditis, and delayed presentation of cardiovascular complications have been investigated [2]; however, at the time of this analysis, there has not been a systematic review and meta-analysis that assessed the prognostic capability of CAC in this patient population.

## 2. Materials and Methods

**Figure 1:**
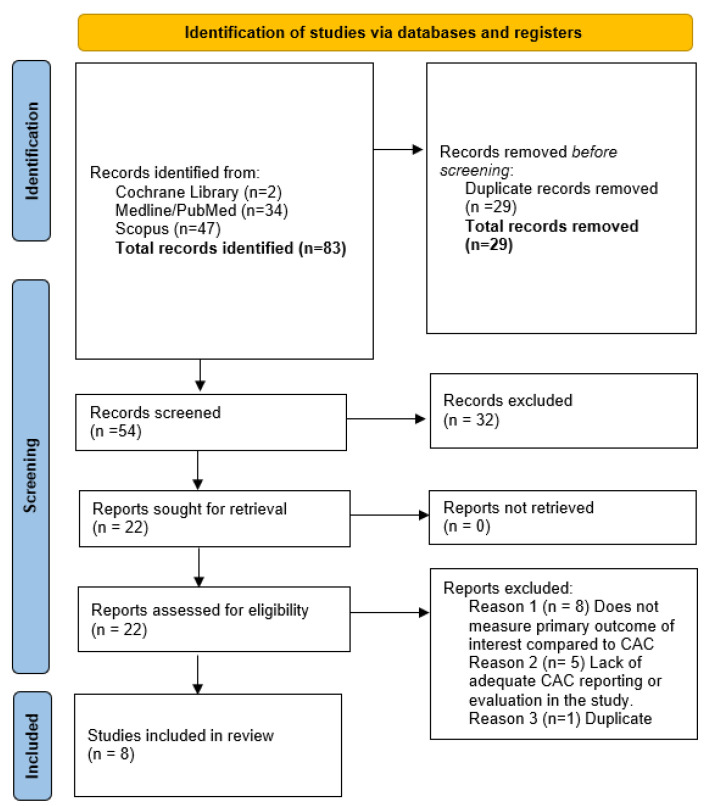
PRISMA flowchart.

The study was registered with the PROSPERO international database. To find relevant studies, search terms included “coronary calcium score” and “COVID-19” as well as other relevant or equivalents terms. A flowchart is outlined as follows in Figure 1 [2].

A study was included if it (1) was an original peer-reviewed study, (2) reported CAC scoring in patients with COVID-19, and (3) investigated ICU admission, mechanical ventilation, and death rate related to individual CAC score in patients with COVID-19. There was no limitation on publication date. Language was limited to English. Review articles, case series, and case studies were excluded. An additional study that met the inclusion criteria was excluded due to an inconsistent data set when internally cross referenced.

Preferred Reporting Items for Systematic Reviews and Meta-Analyses guidelines were applied to data selection. Two authors (K.L. and O.R.) independently assessed the eligibility of each article retrieved with blinding on Rayyan platform [3]. Disagreements were arbitrated by an independent third reviewer.

### 2.1. Data Extraction

Two reviewers (K.L. and O.R.) extracted the following data from included studies: (1) absence or presence of CAC in patients with COVID-19, (2) severity of the CAC, (3) number of ICU admissions, mechanical ventilations, and deaths in patients with COVID-19 with coronary calcifications present on imaging, and (4) number of ICU admissions, mechanical ventilations, and deaths in patients with COVID-19 without coronary calcifications on imaging. The data were compiled in Excel. Extracted data were crosschecked by both investigators (K.L. and O.R.) with the original sources.

### 2.2. Risk of Bias Assessment

Newcastle–Ottawa scale for cohort studies was applied to each included study in the analysis [4]. A composite score between 0 and 9 was calculated based on three categories. Arbitrary values were determined for studies’ risk of bias: high risk 0–3; moderate risk 4–6; and low risk 7–9. A modified scale was used to calculate the score for Dillinger et al. (2020).

### 2.3. Statistical Analysis

Presence of CAC was compared with the absence of CAC based on the interests of outcome. The CAC data were further divided into absent, mild, moderate, or severe CAC in certain studies if it was reported. Absent and mild CAC were determined to be the “low CAC” group, and moderate and severe were stratified into the “high CAC” group. One study utilized the population’s median CAC [5]. This median cutoff determined the “low CAC” and “high CAC” group. In Fazarri et al. (2021), CAC 0–299 was considered as “low CAC” group and above 300 was considered “high CAC” group.

Statistical analysis was performed on Microsoft Excel and PyMeta [6]. A random effects model was applied to the analysis. Presence of CAC and absence of CAC were recorded as well as the associated number of ICU admissions, mechanical ventilations, and deaths. The CAC severity was also recorded in respect to the interest of outcomes. Relative risk ratio and standard error were calculated for each outcome of interest. Inter-quartile ranges were calculated based on 95th percentile range from the aggregate data. Forest plots were used to visualize the analyses. Funnel plot was not applied due to the low number of studies in each outcome. Heterogeneity was assessed based on Cochrane Q score and *I*^2^ index. Cochrane Q *p*-value was calculated based on chi-squared test. Degrees of heterogeneity were based on *I*^2^ indices of 0–25%, 26–50%, 51–75%, and 76–100%, representing very low, low, moderate, and high degrees, respectively.

Based on available data of the original studies, two sets of analysis were performed. Effects of the presence of CAC and absence of CAC were measured. It was further investigated whether or not there was a statistical difference in the outcome based on the severity of the CAC.

## 3. Results

### 3.1. Study Characteristics

A total of eight studies were included in the analysis. Among the included, there were six retrospective cohort studies, one prospective cohort study, and one cross-sectional study. Analysis of these studies involved a total of 4542 individuals. Two studies were in the United States, five in Europe, and one in China. The studies did not have a consensus on method of CAC scoring and the cutoff for severity classification. V-CACS and Agatston scores were utilized in four and four studies, respectively. All studies had a Newcastle–Ottawa scale score between 8 and 9. A modified scale was used to evaluate Dillinger et al. 2020. Table 1 provides summary of the study characteristics, while Table 2 provides raw data from original sources.

### 3.2. Death

**Figure 2:**
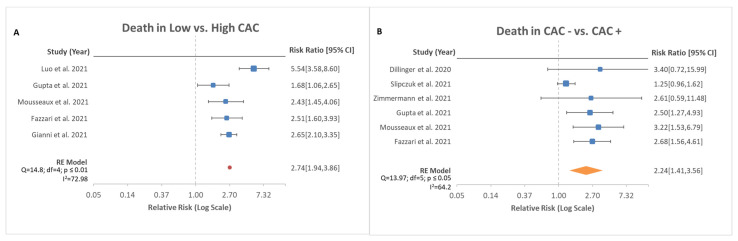
Risk ratio of death based on stratification of CAC scores. (**A**) depicts mortality in low CAC vs. high CAC with total of five studies analyzed with 95% confidence interval. Test for overall effect: Z = 5.71, *p* < 0.00001. (**B**) depicts mortality in absence of CAC vs. the presence of CAC with total of six studies analyzed with 95% confidence interval. Test for overall effect: Z = 3.40, *p* < 0.001.

A total of five studies reported on death rates in patients with COVID-19 with concurrent reported CAC severity in total of 3769 patients. In the pooled analysis, there was an increased risk of mortality with higher CAC score compared with low CAC score (Figure 2A, RR, 2.74; 95% CI, 1.94–3.86; *p* < 0.00001). Further analysis was performed based on the death rates between patients with absent CAC and present CAC in a total of six studies. Luo et al. (2021) was excluded in the latter analysis due to lack of data in patients with absent CAC. There was evidence that CAC increased mortality compared with patients without CAC (Figure 2B, RR, 2.24; 95% CI, 1.41–3.56; *p* < 0.001).

### 3.3. Mechanical Ventilation

**Figure 3:**
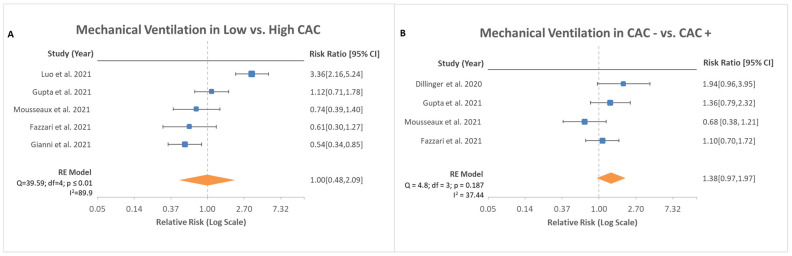
Risk ratio of mechanical ventilation based on stratification of CAC scores. (**A**) depicts risk in low CAC vs. high CAC with total of five studies analyzed with 95% confidence interval. Test for overall effect: Z = 0.00, *p* = 0.99. Test for overall effect: Z = 5.71, *p* < 0.00001. (**B**) depicts risk in absence of CAC vs. the presence of CAC with total of four studies analyzed with 95% confidence interval. Test for overall effect: Z = 0.72, *p* = 0.48.

Mechanical ventilation event rates and severity of CAC were reported in five studies with a reported total of 397 patients out of 3769 patients. Based on the data, no difference was noted between patients with low CAC score and high CAC score in the events of mechanical ventilation (Figure 3A, RR, 1; 95% CI, 0.48–2.09; *p* = 0.999). Further analysis was conducted in four studies for patients with absent CAC and present CAC regarding mechanical ventilation rates. No difference was noted between those two groups as well (Figure 3B, RR, 1.38; 95% CI, 0.97–1.97; *p* = 0.475).

### 3.4. ICU Admission

**Figure 4:**
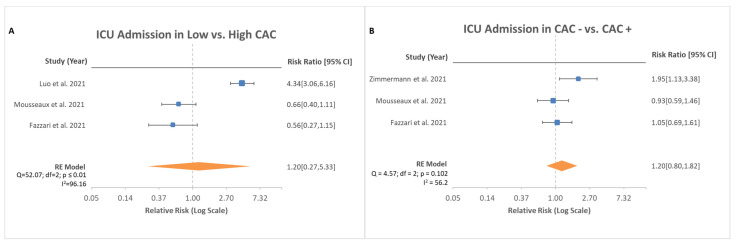
Risk ratio of ICU admission based on stratification of CAC scores. (**A**) depicts risk in low CAC vs. high CAC with total of three studies analyzed with 95% confidence interval. Test for overall effect: Z = 0.23, *p* = 0.82. (**B**) depicts risk in absence of CAC vs. the presence of CAC with total of three studies analyzed with 95% confidence interval. Test for overall effect: Z = 0.88, *p* = 0.38.

Both ICU admission rates and severity of CAC were reported in a total of three studies in a total of 2518 patients. Among the high CAC score group and low CAC score group, there was no significant difference noted (Figure 4A, RR, 1.2; 95% CI, 0.27–5.33; *p* = 0.815). Additional investigation was performed to observe whether rates differed in the presence of CAC and absence of CAC. However, no difference was found between patients with CAC and without CAC (Figure 4B, RR, 1.2; CI 0.80–1.82; *p* = 0.379).

## 4. Discussion

CAC scoring can be defined by multiple methods [1]: Agatston scoring, volume score, mass score, and visualization score (V-CACS). The clinical application of CAC scoring allows delineation and risk stratification of patients with increased atherosclerotic risk [1,7]. CAC score can be obtained by a non-contrast computed tomography of the chest, which may be obtained at the time of COVID-19 diagnosis [8]. Although standardized cutoff values for CAC have previously been studied [1,7], the actual utilized scoring and cutoffs for coronary studies seem user- and institution-dependent. Given the variability in the study methods, this analysis aimed to establish a systematic approach to comparison of CAC scores to synthesize and aggregate data. Few studies utilized V-CACS to separate the patients into absent, mild, moderate, and severe categories [9,10]. Other studies reported scores in Agatston format [5,11,12,13,14]. One study reported as “high CAC” and “low CAC” groups [15]. Based on the provided data, the reviewers classified those data into low CAC and high CAC group. This separation was based on the authors’ opinion, so it may be subjected to observer bias. Objective data on the presence or absence of CAC were analyzed to minimize the risk of observer bias. These two parameters were used to assess risk of three clinical outcomes: death, mechanical ventilation, and ICU admission.

A history of coronary atherosclerotic disease (CAD) is known to be associated with a 10% increased mortality risk in hospitalized patients with COVID-19 [16]. Other studies have reported similar findings. Additionally, COVID-19 has been associated with various cardiovascular complications [17,18]. Hyperinflammation, cytokine storm, lymphohistiocytosis, coronary microvascular thrombosis, and endotheliitis have been proposed to be possible mechanisms of acute myocardial injury in COVID-19 [19]. Slipczuk et al. (2021) measured the epicardial adipose tissue (EAT) and deemed it to be an independent risk factor for mortality in patients with COVID-19. Given the increased oxygen demand as well as cardiovascular strain and stress, patients with a history of CAD are at increased risk for worse clinical outcomes compared with their healthy counterparts. A single-center cohort study showed that there was a lower prevalence of myocardial injury if CAC was absent in patients with COVID-19 [20]. Absence of CAC had a high negative predictive value for major adverse cardiac events in patients hospitalized with COVID-19 even in the presence of other cardiac risk factors [21]. Based on the analysis, the presence of CAC alone increased the mortality rate for hospitalized patients with COVID-19 (Figure 2B). It may be safe to presume that patients with no known history of CAD may benefit from CAC scoring as a prognostic indicator for death during hospitalization.

COVID-19 is a primary respiratory disorder that can lead to acute respiratory distress syndrome (ARDS), which for many patients may require mechanical ventilation [22]. ARDS is defined by noncardiogenic pulmonary edema that can lead to life threatening hypoxia from a lack of proper oxygen exchange within the lung parenchyma [23]. The therapy for ARDS in COVID-19 is multidisciplinary, but the mainstay for critically ill patients is low tidal volume mechanical ventilation [23]. The risk of being mechanically ventilated is based on a multitude of factors such as age, obesity, cardiovascular disease, and socioeconomic background [24]. In a single-center cohort study of 5279 patients, the strongest risk factor for development to severe illness and possible intubation, in addition to age, was heart failure, with an odds ratio of 1.9 [24]. This analysis looked to assess whether CAC was a possible risk factor for intubation and mechanical ventilation. Of the studies reviewed, Luo et al. (2021) noted an increased risk for mechanical ventilation with high CAC scores; however, the aggregate data were not consistent with this finding. All other studies that were reviewed found a lack of significant elevation in risk for mechanical ventilation related to CAC levels. These data suggest that CAC presence and severity is not an independent risk factor for mechanical ventilation in hospitalized patients with COVID-19.

Hospitalized patients with COVID-19 are at increased risk for multi-organ systemic disease. A study of 138 patients who developed critical illness found the median time from admission to critical care service to be 2.5 days after onset of symptoms [25]. This rapid deterioration can be attributed to the severe pulmonary insult from the infection, but many of these patients have other non-pulmonary complications that lead to ICU admission. The complications most notably found during these admissions include acute kidney injury, encephalopathy, thrombosis, and cardiac injury [24]. Cardiac complications have ranged from cardiomyopathy and arrythmia to myocardial infarction and cardiac arrest [26,27]. One single-center cohort found a 33 percent occurrence of cardiomyopathy in critically ill patients with COVID-19 [28]. This cardiomyopathy is primarily thought to be either related to the hypercoagulable state from increased inflammation leading to coronary thrombosis and ischemic heart disease or direct cardiac myocyte injury from the viral pathogen [29]. However, the contribution of each of these complications to major adverse cardiovascular outcomes has not been fully determined. In a study examining 700 critically ill patients with COVID-19 with arrhythmias, nine patients developed cardiac arrest, all of whom were in the ICU. The development of cardiac arrest was associated with acute in-hospital mortality [30]. With a short median time from admission to an ICU transfer, as well as the known cardiovascular complications that follow, early prognosis via CAC scoring may benefit to risk-stratify patients. As seen in Figure 3A, there was no significant difference noted between high and low CAC scores for ICU admission risk. There was also no significant difference noted in ICU admission risk whether any CAC was found. Given these findings, the risk of being admitted to an ICU for hospitalized patients with COVID-19 appears to be multifactorial in nature but independent of CAC scoring.

### Limitations of the Study

This analysis reviewed eight studies on CAC and COVID-19 hospitalization but was not without its limitations. One such limitation was that in the collecting of the individual study data, the scoring of CAC via V-CACS is dependent on the operator who reviewed the CT images, therefore leading to observer bias. However, the studies minimized such bias by employing two independent reviewers. Even in studies with Agatston scores, the cutoff scores varied depending on the study. Additionally, there was no consensus on method of CAC scoring. However, the ordinal method of CAC scoring correlated well with the Agatston score in terms of predicting cardiovascular disease [31,32,33]. As such, the analysis between the prognosis difference of V-CACS and ordinal scoring was not pursued in this study. Nonetheless, it may be a topic of interest for future research in the setting of COVID-19. Furthermore, although eight studies were analyzed, not every study included data on ICU admission or risk of mechanical ventilation.

## 5. Conclusions

This meta-analysis strictly examined the outcomes of interest in death, mechanical ventilation, and ICU admission while comparing the CAC scores in patients with COVID-19. The compiled data show that CAC was associated with mortality increase by approximately 2 folds. However, the exact mechanism of why CAC increases mortality rate is uncertain. It may be due to myocarditis, type 1 myocardial infarction, type 2 myocardial infarction, or other causes such as increased risk of cardiac arrest. Coronary atherosclerosis is known to be a significant risk factor for poor outcomes in COVID-19 hospitalizations. Patients with no known history of CAD can be non-invasively assessed for possible coronary atherosclerosis via CAC scoring. CAC scoring may not correlate directly with risk of mechanical ventilation or ICU admission in this patient population. Given these findings, CAC scoring can aid in stratifying patients, thus allowing earlier interventions in rapidly developing illnesses.

## Figures and Tables

**Table 1:** Summary of study characteristics.

Study	Type of Study	Region	CAC Scoring	Events Reported	Newcastle-Ottawa Scale
Luo et al. 2021	Retrospective Cohort	China	V-CACS	Death Mechanical Ventilation ICU Admission	9
Gupta et al. 2021	Retrospective Cohort	United States	V-CACS	Death Mechanical Ventilation ICU Admission	9
Mousseaux et al. 2021	Prospective Cohort	France	V-CACS	Death Mechanical Ventilation ICU Admission	8
Fazzari et al. 2021	Retrospective Cohort	Italy	Agatston	Death Mechanical Ventilation ICU Admission	9
Gianni et al. 2021	Retrospective Cohort	Italy	Agatston	Death Mechanical Ventilation	8
Dillinger et al. 2020 *	Cross-sectional	France	Agatston	Death Mechanical Ventilation	8
Slipczuk et al. 2021	Retrospective Cohort	United States	V-CACS	Death	9
Zimmermann et al. 2021	Retrospective Cohort	Germany	Agatston	Death ICU Admission	8

* Required modified Newcastle–Ottawa scale due to its study type.

**Table 2:** Individualized data of each study.

Study	CAC Severity	Total Patients (N)	Death	ICU Admission	Mechanical Ventilation
Luo et al. 2021	High CAC	177	27	37	23
	Low CAC	1890	52	91	73
Gupta et al. 2021	Absent	51	8	--	13
	Mild	42	15	--	15
	Moderate	23	8	--	10
	Severe	42	19	--	12
Mousseaux et al. 2021	Absent	64	7	21	17
	Mild	42	11	17	8
	Moderate	26	9	8	4
	Heavy	37	17	7	7
Fazzari et al. 2021	CAC 0	138	15	31	28
	CAC 1–299	94	22	27	25
	CAC 300–999	34	12	4	4
	CAC ≥ 1000	16	8	3	3
Gianni et al. 2021	CAC ≤ 335.48	868	125	--	136
	CAC > 335.48	225	86	--	19
Dillinger et al. 2020	CAC = 0	103	2	--	10
	CAC ≥ 1	106	7	--	20
Slipczuk et al. 2021	CAC = 0	147	50	--	--
	CAC ≥ 1	308	131	--	--
Zimmermann et al. 2021	CAC = 0	40	2	11	--
	CAC ≥ 1	69	9	37	--
Total	--	4542	642	294	427

## Data Availability

Not applicable.
